# Presence of protein production enhancers results in significantly higher methanol-induced protein production in *Pichia pastoris*

**DOI:** 10.1186/s12934-018-0961-4

**Published:** 2018-07-13

**Authors:** Loknath Gidijala, Stefan Uthoff, Sebastiaan J. van Kampen, Alexander Steinbüchel, Raymond M. D. Verhaert

**Affiliations:** 1 ProteoNic BV, J.H. Oortweg 19-21, 2333 CH Leiden, The Netherlands; 2 Institut für Molekulare Mikrobiologie und Biotechnologie, Westfälische Wilhelms-Universität Münster, Corrensstraße 3, 48149 Münster, Germany; 3 Present Address: Hubrecht Institute, KNAW and University Medical Center Utrecht, Uppsalalaan 8, 3584 CT Utrecht, The Netherlands; 4 Environmental Sciences Department, King Abdulaziz University, Jeddah, 21589 Saudi Arabia

**Keywords:** Recombinant protein production, Yield enhancement, *Pichia pastoris*, Yeast, Gene expression

## Abstract

**Background:**

The yeast *Komagataella phaffii*, better known as *Pichia pastoris*, is a commonly used host for recombinant protein production. Here expression vectors are reported that address the different steps of the transcription–translation–secretion pathway of heterologous protein production.

**Results:**

Transcription and translation enhancing elements were introduced in an expression cassette for the production of recombinant *Aspergillus niger* feruloyl esterase A. The yield was increased by threefold as compared to the yield without these elements. Multiple copy strains were selected using a zeocin resistance marker in the expression cassette and showed another sixfold higher yield. Modification of the C-terminal amino acid sequence of the secretion signal did not significantly improve the production yield. Similar data were obtained for the production of another protein, recombinant human interleukin 8. Upscaling to fed-batch fermentation conditions resulted in a twofold increase for reference strains, while for strains with enhancing elements a tenfold improvement was observed.

**Conclusions:**

*Pichia pastoris* is used for recombinant protein production in industrial fermentations. By addressing the transcription and translation of mRNA coding for recombinant protein, significant yield improvement was obtained. The yield improvement obtained under microscale conditions was maintained under fed-batch fermentation conditions. These data demonstrate the potential of these expression vectors for large scale application as improved production of proteins has major implications on the economics and sustainability of biocatalyst dependent production processes e.g. for the production of pharmaceuticals and for the bioconversions of complex molecules.

## Background

The methylotrophic yeast *Pichia pastoris* is known as an excellent host for recombinant protein production [1–3]. Two easy-to-use expression systems are commercially available for *P. pastoris.* One is based on the methanol induced AOX1 promoter. The other exploits the constitutively expressed GAP promoter, which has a key role in glycolysis. The methanol inducible expression system is often preferred over the constitutive expression as the growth phase can be decoupled from the protein production phase. Methylotrophic yeast can convert methanol to energy with a key role of the first enzyme, alcohol oxidase (AOX1P) in the methanol catabolic pathway. The promoter of alcohol oxidase (P*AOX1*) is tightly regulated by both *cis*- and *trans*-regulatory elements (Table 1 and [4]). Methanol acts as a catabolite inducer of P*AOX1* which is repressed when cells are grown on glucose as carbon source. Upon switching the carbon source to methanol it first becomes derepressed and subsequently induced [4]. The transcription factor Mxr1p was designated as a key regulator for P*AOXI* induction and targeted deletions have resulted in P*AOX1* libraries that enable to vary the expression level [5]. Other studies demonstrated that insertion of three copies of the transcription factor Adr1P binding region upstream of P*AOX*1 resulted in 50% increase in P*AOX*1 expression [6]. Heterologous enhancers can also be used to improve the protein production: Insertion of the polyoma enhancer upstream of the *His*3 promoter was shown to enhance the *His*3 expression [7].

**Table 1:** Summary of *Pichia pastoris* alcohol oxidase promoter regulatory elements

Regulatory elements	Description	Classification	Refs
*Mxr*I	Transcription factor involved in the activation of the methanol metabolism genes	*Cis*	[31]
Region-D	Transcription factor binding site involved in the binding of the transcription factor AdrIp	*Cis*	[6]
Putative TFBS regions	AOX1 promoter library generated by systematic deletion of sequences within the P*AOX*1	*Cis*	[5]
Protein 14.3.3	Binds to Mxr1P and further inhibits all Mxr1p regulated gene repression	*Trans*	[32]
Prm1	Constitutive expression of the PRM1 results in activation of the P*AOX*1 in methanol free medium	*Trans*	[33]
Mit1	MIT1 regulates methanol induction of P*AOX*1	*Trans*	[34]
HXT1	Pp HXT1 is necessary for catabolite repression of P*AOX*1	*Trans*	[35]
ZTA1	Single strand DNA binding protein, which regulates P*AOX*1 during oxidative stress	*Trans*	[36]
5′ UTR	4-nucleotide sequence immediately preceding AUG start codon A-rich	*Kozak sequence*	[10]
P*AOX*1-5′ UTR	Several regions identified affecting translation positively as well as negatively	*Cis*	[14]

*Cis* regulatory elements are regions within the P*AOX*1 that act as a transcriptional factor binding sites, *Trans* regulatory elements are regulatory proteins/transcriptional factors that bind to the cis regulatory regions

In most applications of *Pichia* to produce heterologous protein, the foreign gene of interest is inserted between the 5′UTR and 3′UTR of the *Aox1* gene. Several roles have been attributed to the 5′UTR of genes in *Pichia* and other organisms regarding stability and translational efficiency of the mRNA [8]. The 5′UTR of the mRNA of most proteins in yeast is short (< 100 bps) and low in GC% [9]. The sequence directly upstream of the AUG start codon is most important: The relationship between 2041 5′UTR variants from *Rpl8A* and protein synthesis efficiency indicated that a purine located 3 nucleotides upstream (− 3) of the AUG resulted in improved protein synthesis as compared to variants without a − 3 purine. Similarly a genome wide survey of highly expressed genes showed that natural 5′UTRs in yeast are typically “A”-rich, particularly at positions − 4 to − 1 of the AUG start codon [10, 11].

Transcriptome analyses of *Pichia* using RNA sequence analysis indicate that the median length of *P. pastoris* 5′UTR (102 bps) is significantly larger than the median, 50 bps 5′UTR length of *S. cerevisiae* [12, 13]. The 5′UTR of AOX1, the most used promoter for heterologous protein expression in *Pichia* has a length of 114 bps. It contains regions that affect translation in both a positive and a negative manner [14]. Also changes in the 5′UTR which are not located in the Kozak sequence affect the production yield [15, 16]. Therefore, replacing the AOX1 5′UTR by other sequences may improve heterologous protein production yield.

Production of recombinant proteins has also gained attraction for its role in replacing chemical conversion by bioconversions. Indeed, increasing criticism concerning the utilization of chemical processes for the synthesis of small sugar and phenolic moieties triggers an increasing demand for eco-friendly degradation or bioconversion of lignocellulose. However, with respect to the production scale and costs, breakdown or conversion of lignocelluloses by esterases cannot compete yet with chemical conversions, as the enzyme costs are too high. Therefore, there is a need for an expression system that can efficiently secrete functional recombinant protein for both developing enzymes with new properties as well as for producing them cost-effectively. *Pichia* has the advantage that it secretes only a small amount of endogenous proteins and a more efficient *Pichia* expression system allows economical screening for new and modified glycosylated enzymes.

The goal of this study was to explore the effect of combining different genetic elements affecting protein production yield. Two elements were placed upstream of the AOX1 promoter and combined with a sequence replacing the native AOX1 5′UTR. Performance studies with both single and multiple copy stains were carried out, the latter being especially important for industrial application. Also the performance of these elements in combination with the different signal peptide sequences used to secrete heterologous proteins was verified. Fed batch bioreactor runs of the strains explored the use and value of the elements after scale-up.

## Results and discussion

### Effect of protein production enhancing elements on FAE protein production

**Fig. 1:**
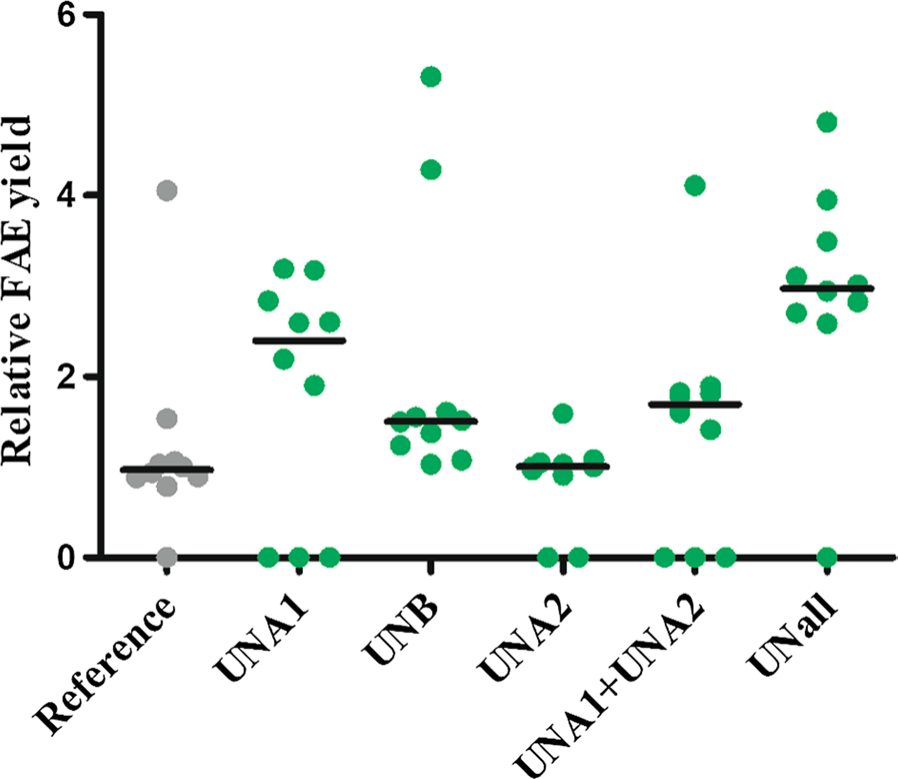
Protein yield represented relative to median value of reference strains. Effect of putative transcription and translation enhancing elements on the production yield of feruloyl esterase (FAE) by *Pichia pastoris* GS115. Microtiter scale protein production yield of ten transformants selected by histidine complementation after 72 h of methanol induction in BMMy. FAE protein yield was quantified by the specific rate of the enzyme solution to hydrolyse 4-nitrophenyl ferulate. Dots represent production data from individual transformants. To compare element and element combinations performance median yield values are used which are indicated by a horizontal line. UNA1 and UNA2 represent the regulatory elements upstream of the P*AOX*1, UNB represents a modified 5′UTR. UNall represent the combination of regulatory elements UNA1, UNA2, and UNB

In this study, three different elements were used to obtain production yield improvement: UNA elements and UNB were hypothesised to affect transcription and translation, respectively. A series of expression vectors carrying these elements was generated to determine the effect on methanol-induced protein production of recombinant feruloyl esterase (FAE). Besides the FAE amino acid coding sequence, the nucleotide sequence in the expression cassette of the reference constructs was identical to the one used in commercial vectors, including its secretion signal (e.g. pPIC9 series). Regulatory sequences and combinations thereof were compared with the reference sequence, leading to a set of six expression vectors. Independent transformants for each of the six vectors were isolated by selection for histidine biosynthesis complementation. In general this method results in the isolation of clones of which > 90% carry only one copy of the expression cassette integrated into the genome [17]. Methanol induced protein production was carried out in complex medium (BMMy) and supernatants of the production medium were collected after 72 h to determine the FAE production yield. This was accomplished by using an assay for the FAE catalysed hydrolysis of 4-nitrophenyl ferulate in which a linear relationship exists between the amount of FAE and the conversion rate (see “Methods” section). The production yields—six different constructs, ten strains each—are presented in Fig. 1. Within each series of strains, significant differences between the expression levels were observed: on one hand, one or two of the transformants independent of the expression vector type outperforms the other transformants by up to fivefold and on the other hand a number of selected transformants do not produce a significant amount of FAE. These observations are in line with literature data and attributed to multiple copy insertion of the expression cassette and to non-integrative recombination between the *his*4 gene on the chromosome and the *his4* gene on the plasmid, respectively [17–19]. Therefore the median value of the FAE production levels, which represents the production by clones harbouring a single, properly inserted copy of the expression cassette, is used to compare the effect of the UNA and UNB elements on FAE production.

The production data indicate that the expression vector containing only UNA2 did not result in any improvement, while the presence of all other vectors containing other single elements or combinations of UNA and UNB elements lead to a 1.5 to threefold higher protein yield (Fig. 1). Interestingly, despite any yield improving effect of UNA2 if present as a single element, the combination of UNA1 and UNA2 did result in an improvement which was similar to the improvement by UNB. This suggests that the use of enhancers does not result in an additive effect on protein production improvement. The expression cassette with UNA1, UNA2, and UNB results into the best transformants for production of FAE.

### Increasing gene dosage of *faeA* expression cassette confirms single copy results

Single copy transformants with the combination of UNA1, UNA2 and UNB, now named UNall, produce threefold more FAE than transformants without these elements. In most applications however, instead of single copy strains, strains with multiple copies of the gene of interest are used because the protein yield increases upon increasing the gene dose in the strain.

Most commonly multiple copy strains are made by zeocin resistance selection. The reason for this is that selection of transformants on increasing levels of zeocin results in transformants carrying an elevated number of copies by a post transformational modification process [20, 21]. The precise relationship between production yield and (zeocin-induced) copy number depends on the type of protein, e.g. for one protein a maximum was reached with three copies (Na-ASP1 protein from *Necator americanus* [22]), while for another only after integration of 12 copies a maximum production was reached (porcine insulin precursor [23]). Therefore, to obtain the highest number of copies, the zeocin concentration in this study was varied till its possible maximum. Thus, strains were isolated after either selection for histidine complementation or zeocin selection and their production yield determined.

**Fig. 2:**
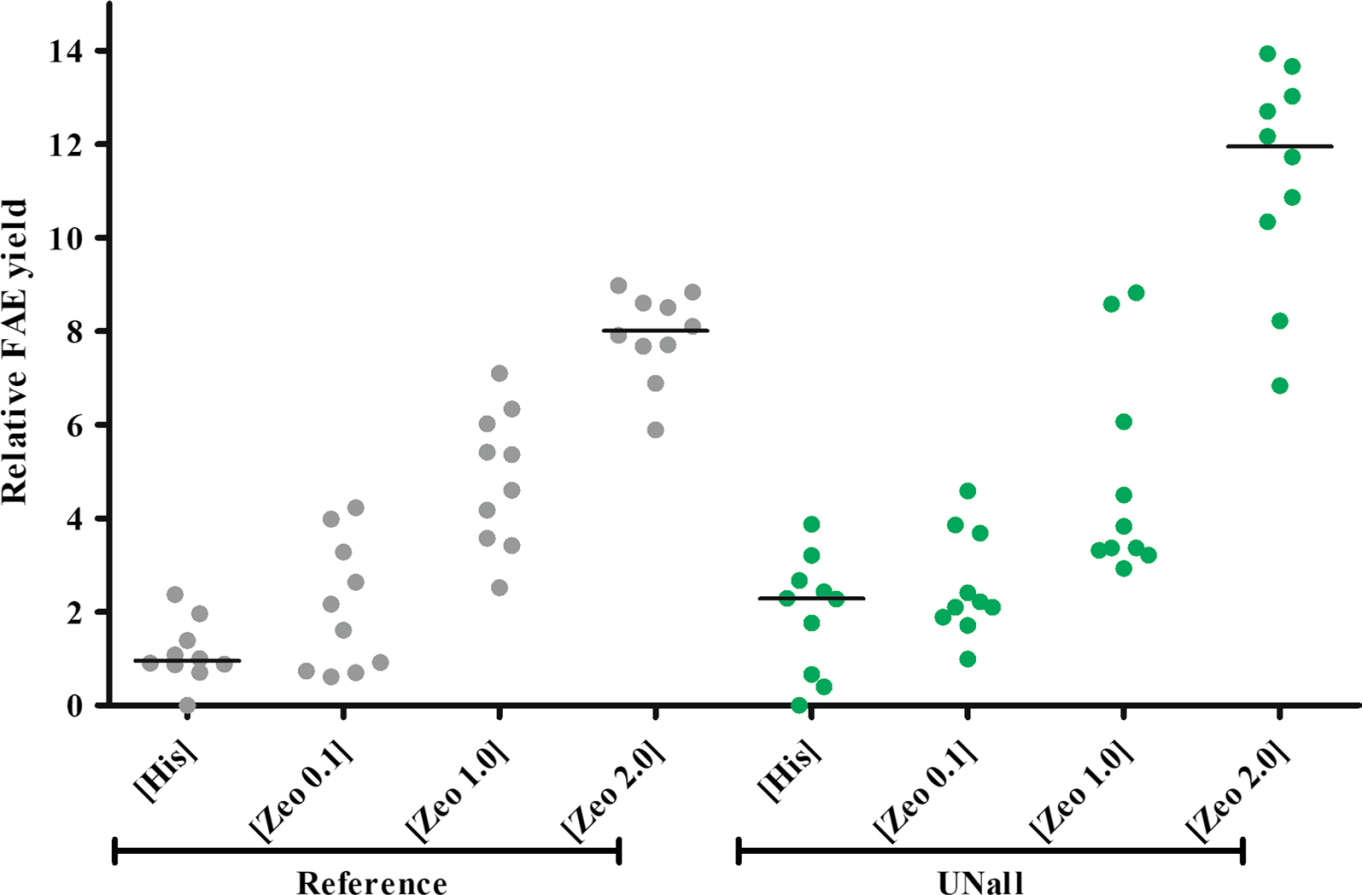
FAE production yields of histidine and zeocin selected reference and UNall strains. Reference strains (grey) or UNall strains (green) were selected either by histidine complementation or on 0.1, 1.0 or 2.0 mg/mL zeocin. Productivity was determined by microscale fermentation

The relative FAE production yield observed for histidine complemented, single copy reference strains was set to 1.0. The yield of the UNall strains was 2.4 (Fig. 2). Similar to the data in Fig. 1 this represents a difference of approximately twofold. The strains selected at 0.1 mg/mL zeocin produce similar amounts of FAE as the strains selected by histidine complementation, indicating that by selection at low zeocin concentration mainly single copy transformants are isolated.

Strains could be isolated from plates with a zeocin concentration ranging between 0.1 and 2.0 mg/mL. At a higher concentration of zeocin no transformants were obtained. As expected and in accordance with literature data, strains isolated after transformation and selection on increased concentrations of zeocin display an increased FAE production yield: the higher the concentration of zeocin on which the strains were selected, the higher production of the isolated strains (Fig. 2). The yield of the strains with the reference construct increases from 1.0 (single copy) to 7.9 (the best strain, 2.0 mg/mL zeocin), while for the UNall strains the increase is from 2.2 to 12.2. Indeed, this relationship between the zeocin selection concentration and the protein yield indicates that the yield increases upon an increase of the zeocin harbouring expression cassette. Thus, zeocin-selected transformants produce up to eightfold more protein than the transformants typically obtained by histidine complementation (Fig. 2). Remarkably, the production yield does not plateau upon increasing the zeocin concentration to 2 mg/mL. Since no strains survived a concentration exceeding that amount of zeocin, it is not clear whether the currently produced amount of FAE under these conditions represents a maximum. Importantly, the FAE production yield data between the strains with and without UNall differ by a factor of two both at the single copy level as well as on the level obtained during zeocin selections. This indicates that at all conditions the UNall harbouring strains maintain their production yield advantage relative to the reference strains.

### Effect of α-mating factor spacer peptide on recombinant protein production

The introduction of protein production enhancing elements (PPEs) results in a yield improvement in both single and multiple copy expression. Due to the extraordinary potent secretory system of *Pichia* strains this yeast is predominantly used for the production of secreted proteins, helping to enhance protein downstream processing efforts. For the secretion process, most *Pichia* expression systems use the *Saccharomyces* α-mating factor pre-pro leader sequence (MFA) to obtain efficient secretion of recombinant protein into the culture supernatant. This MFA secretion signal has a C-terminal sequence of Ser-Leu-Glu-Lys-Arg (SLEKR) followed by a Glu-Ala-Glu-Ala (EAEA) spacer dipeptide repeat [24]. *Pichia* efficiently processes the MFA secretion signal in the endoplasmic reticulum by Kex2p endopeptidase, which recognizes and cleaves the signal peptide between the lysine and arginine residue. Subsequently, the EAEA repeat is cleaved as dipeptides by the Ste13 protein in the Golgi vesicles [25]. The addition of extra amino acids downstream of the kex2P recognition site (SLEKR) is reported to result in a production yield improvement of recombinant protein [24]. The C terminal sequence of the MFA during the development of UNall, described in the previous sections, was SLEKR-EAEAYVEF. It is one of the sequences used in commercially available expression vectors. Here the performance of UNall in combination with EAYVEF and EAEA terminal sequences is measured. These C terminal spacer sequences are also used in industrial fermentation processes.

**Fig. 3:**
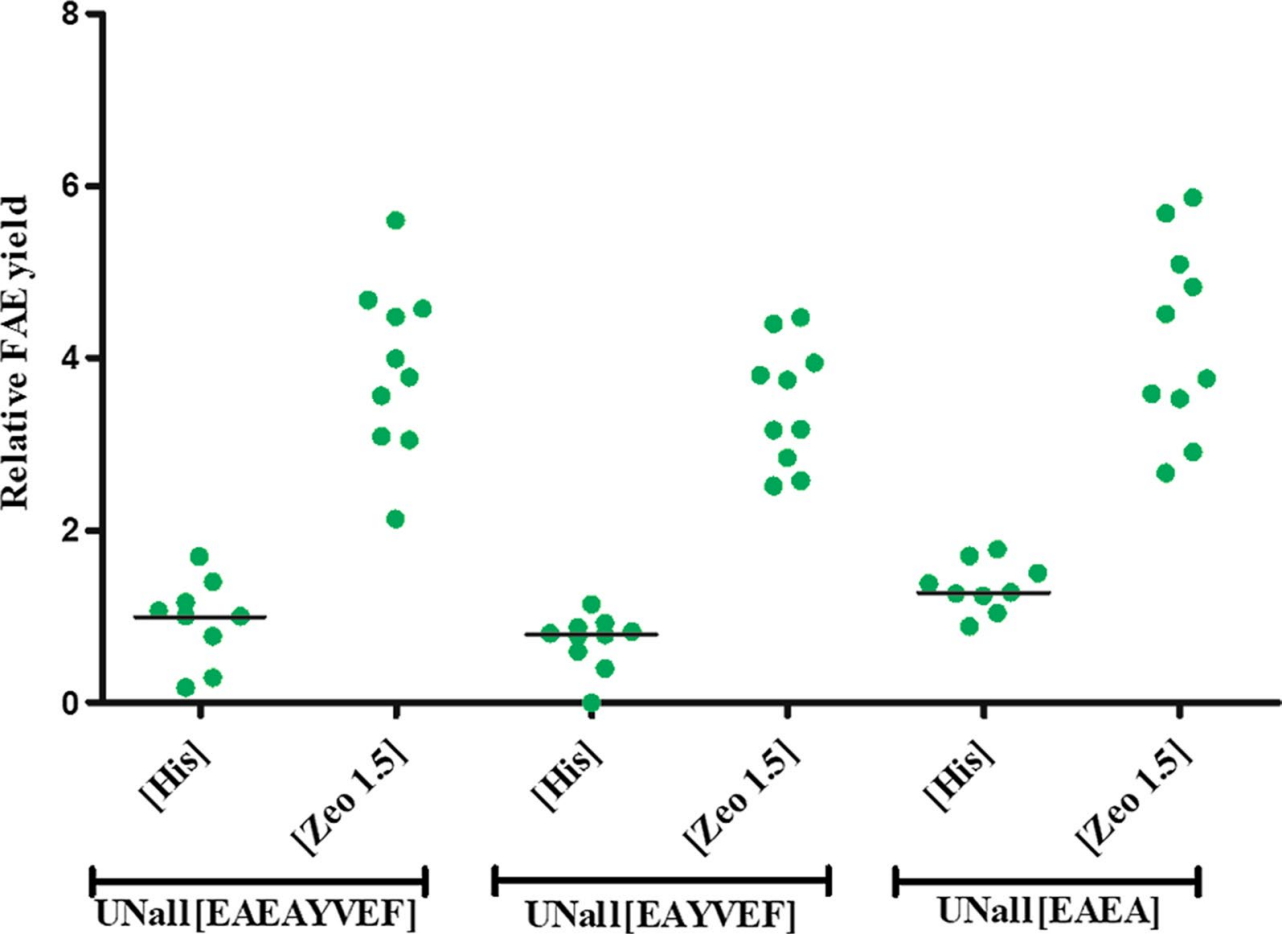
Effect of MFA spacer peptide variation on FAE yield in strains obtained by His complementation or zeocin selection. The single-letter amino acid coded spacer peptide sequence is provided in brackets in the graph

Transformants expressing FAE using UNall and EAEAYVEF, EAYVEF or EAEA sequences were selected by either histidine complementation or zeocin selection. Their productivity was tested by microtiter plate fermentation as described in the “Methods” section. The results indicate a difference between the single copy and multiple copy strains: Though the signal sequence does affect the yield in single copy transformants—the C-terminal sequence EAEA results in a factor of two higher production yield, the yield for multiple copy number transformants is similar for all three C-terminal peptide variants (Fig. 3). This similar yield enhancement of recombinant production and secretion implies that the enhancing elements can be applied in combination with different MFA spacer peptides in industrially relevant, multiple copy strains.

### Application of genetic enhancers for protein production improvement of interleukin 8

Besides FAE production, also the expression of human interleukin 8 (hIL8) using UNall was measured. IL8 is a member of the CXC chemokine subfamily and produced by e.g. epithelial and blood cells. It is commonly known as chemoattractant for neutrophils and it is valued for its use as a diagnostic marker [26]. The effect of UNall on IL8 production was compared with its effect on FAE production to determine whether the UNall enhancing effect is independent from the expressed protein. Strains with reference hIL8 expression cassettes and strains with expression cassettes equipped with UNall were prepared both by histidine complementation and by zeocin selection. Similar to the FAE production, hIL8 production yields of UNall transformants were twofold higher than the yields of the reference strains (data not shown).

**Fig. 4:**
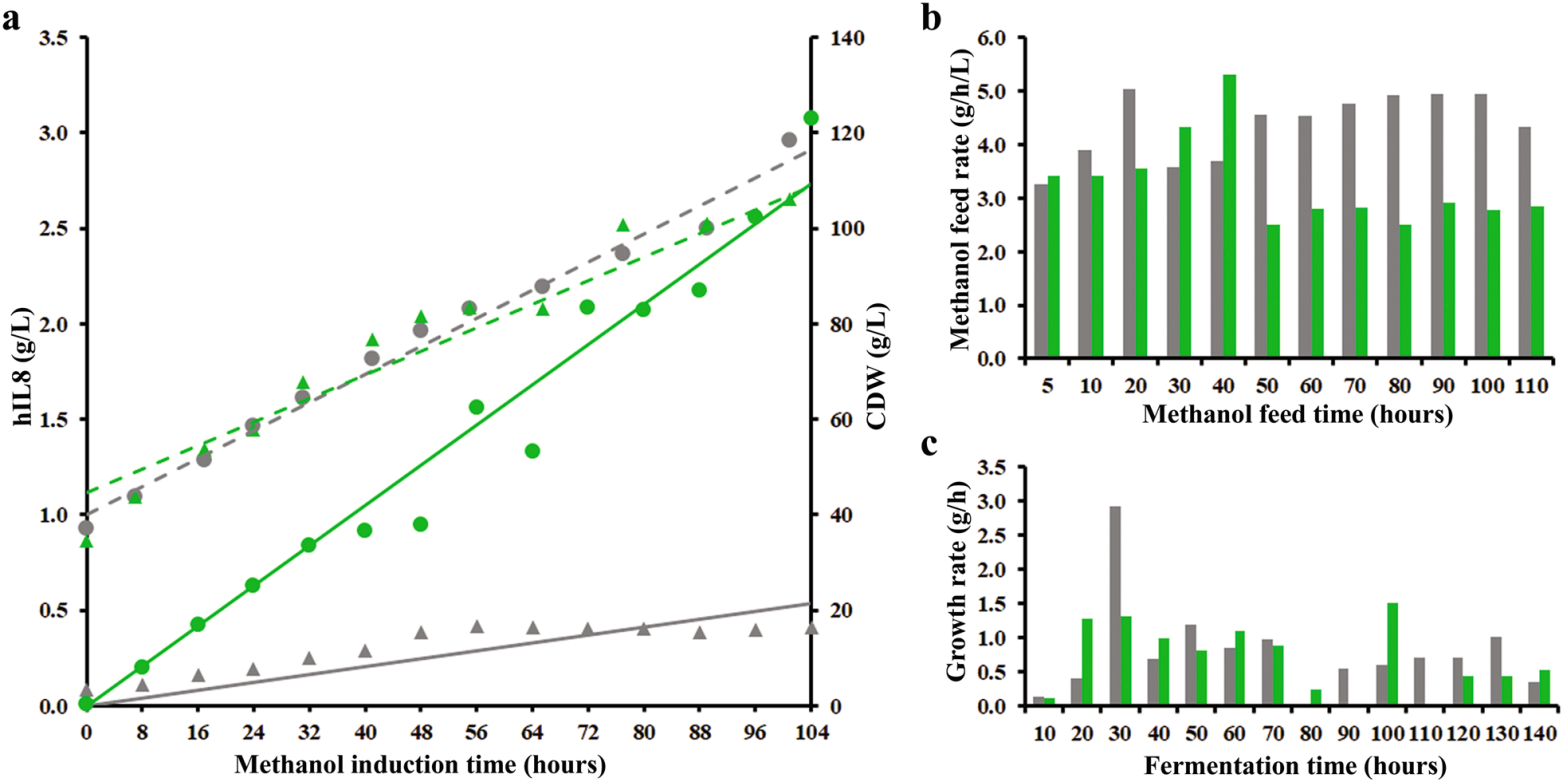
16 L Bioreactor methanol-induced fermentation of interleukin 8 (IL8) by *Pichia pastoris*: **a** Chronological development of cell dry weight (CDW, dashed lines) and secretion of IL8 into the production medium (solid lines). Reference strain data are in grey; PPEs strain data are in green. **b** Methanol feed rate after induction start. For calculation of CDW and methanol feed rates, see “Methods”. **c** CDW growth rate. Switch from glycerol to methanol made after 31–33 h initial growth

In order to verify that the protein production enhancement observed by using PPEs in small scale production conditions is maintained at controlled large scale fermentation conditions, the best hIL8 producing strains with UNall (small scale IL8 yield 0.30 g/L) and without UNall (small scale IL8 yield 0.18 g/L) were cultivated in a 30 L Biostat UD-30 stainless steel bioreactor at 16 L scale. After initial growth with glycerol the feeding of both strains was switched to methanol. The expression of the target protein was induced after about 31 h (reference strain; Ref-IL8) or 33 h, respectively (PPEs containing strain; PN-IL8) at comparable cell dry weights (CDW) of about 43 g/L each. Although the reference strain reached a ~ 10% higher final CDW than the PN-IL8 strain, the CDW data indicate an comparable and continuous cell growth with methanol in both the Ref-IL8 and the PN-IL8 runs suggesting high cell viability (Fig. 4a). The slightly higher final CDW of the Ref-IL8 strain was probably caused by alterations within the metabolism of both strains indicated by the observation that after about 40 h post induction significantly lower methanol feed rates had to be applied to the PN-IL8 culture to maintain a sufficient O_2_ saturation in the medium while the reference strain could be even fed at slightly higher methanol feed rates (Fig. 4b). As expected, different feed rates also resulted in changed CDW growth rates. After about 40 h post induction (i.e. 70–80 h of total cultivation time) growth rates of the PN-IL8 strain commonly decreased to 0–0.5 g/h/L while the Ref-IL8 strain still grew with rates of 0.5–1 g/h/L (Fig. 4c), finally resulting in the mentioned 10% higher CDW of the reference strain at the moment the fermentation was stopped. Generally, differences in fermentation parameters like pO_2_, stirring rate or anti-foaming agent usage may lead to changed methanol consumption rates. Since those parameters, as well as pH and temperature, were kept at comparable levels or stable in both fermentation experiments and anti-foam usage was limited to an absolutely necessary minimum after induction, most likely strain specific differences like mRNA levels or gene copy numbers have influenced the methanol utilization behaviour of the strains as was already described earlier for *P. pastoris* by Zhu et al. [27]. Maximum IL8 production rate in the Ref-IL8 was reached after 48 h of methanol induction and the protein yields remained at that level till the end of the fermentation. Unlike the reference strain, hIL8 production by the strain with PPEs (PN-IL8) is significantly higher. Interestingly, the protein production of PN-IL8 cells after 31 h of methanol induction resulted in a large increase in hIL8 protein production. Moreover, it continued to increase till the end of the fermentation, resulting in a hIL8 concentration of about 3 g/L, thus, representing a tenfold improvement in hIL8 protein production using the PPEs containing *Pichia* strain (PN-IL8) if compared to the reference strain (Ref-IL8). Scaling up the cultivations from a deep well microtiter plate to a controlled bioreactor resulted in a relative yield increase by the PPEs from approx. two-fold in deep well plates to over tenfold in a fermenter. These results show that the yield improving effect of PPEs detected in microtiter plate protein production fermentations are at least maintained in a scaling up process to a significantly larger volume and that the positive effects on protein production can be multiplied if enhanced *Pichia* strains are grown under the controlled and more stable conditions possible with bioreactor based cultivations.

## Conclusions

Protein production was studied by the use of regulatory elements UNA1, UNA2 and UNB. The combination of three elements resulted in the highest yield. Integration of UNall, the combination of UNA1, UNA2 and UNB, in the expression cassette results in improved protein yield of recombinant expression *A. niger* FaeA and human IL8 in *Pichia*. The improved expression cassette resulted in a twofold higher recombinant *A. niger* feruloyl esterase yield. The improvement was similar when combined with different MFA signal sequences in multiple copy strains. Scaling up the production of *Pichia* without UNall from a microtiter plate to a controlled bioreactor resulted in a twofold yield increase. However, in the presence of UNall, under these industrially relevant conditions the yield difference between the reference and UNall increased to almost eightfold. Therefore, the application of UN-elements for recombinant protein production with *Pichia* can serve as a novel tool to increase recombinant protein yields.

## Methods

### Microorganisms and growth conditions

*Pichia pastoris* strain GS115-*his*4 [Invitrogen] was grown at 30 °C in either (i) rich, complex medium (YPD) containing 1% yeast extract, 1% peptone and 1% glucose, (ii) selective medium containing 1.34% yeast nitrogen base without amino acids (Sigma) supplemented with 1.0% glucose (15YND), (iii) Buffered minimal medium (BM) containing 1.34% yeast nitrogen base without amino acids, 100 mM potassium phosphate buffer pH 7.0, as carbon source 1% glycerol (BMG) or 0.5% methanol (BMM), or (iv) Buffered complex medium containing 1% yeast extract, 2% peptone, 1.34% yeast nitrogen base and 100 mM potassium phosphate buffer pH 7.0, containing 1% glycerol (BMGy) or 0.5% methanol (BMMy) as carbon source. For growth on plates, 2% granulated agar was added to the media.

Sub-cloning efficient competent *Escherichia coli* DH5-α (Thermo Fisher Scientific) were used for all cloning purposes. It was grown at 37 °C in LB medium (1% bacto-tryptone, 0.5% yeast extract, 0.5% NaCl) supplemented with 50 mg/L ampicillin, 50 mg/L kanamycin or 25 mg/L zeocin when required.

### *P. pastoris* transformation and screening

*Pichia pastoris* GS115 (*his*4) was grown in 250 mL of YPD for 18 h and prepared for transformation [28]. Two microgram of DNA (Table 2) was linearized by addition of *KspA*I (Thermo Fisher Scientific) and used to transform *P. pastoris* by electroporation (Eppendorf Eporator^®^) set at 1.5 kV using 2 mm gap electroporation cuvettes. The electroporated cells were immediately diluted in 1 mL YPD and after 1 h incubation at 30 °C, 200 µL of the cell suspension was spread either on YND agar plates (without amino acids) or on YPD agar plates containing zeocin. Plates were incubated at 30 °C. Typically, recombinant colonies became visible after 72 h of incubation. For screening purposes, 20 transformants selected on the appropriate plates were streaked on YND plates to confirm histidine complementation. Ten histidine-complemented clones per each expression vector were used for microliter cultivation.

**Table 2:** List of expression plasmid used in this study

Plasmid	Description	Short name construct	Gene of interest	Reference
pPICZαA	-*/PAOX/5′UTR/MFA/TAOX; Zeo*^*R*^		–	b
pPIC9Kα	-*/PAOX/5′UTR/MFA/TAOX; his4, Kan*^*R*^		–	b
pPNic384	-*/PAOX/5′UTR/MFA/TAOX; his4, Kan*^*R*^		*IL8*	a
pPNic775	*UNA1/UNA2/PAOX/UNB/MFA/TAOX; his4, Kan* ^*R*^		*IL8*	a
pPNic948	-*/PAOX/5′UTR/MFA/TAOX; his4, Zeo*^*R*^	*Ref*	*IL8*	a
pPNic947	*UNA1/UNA2/PAOX/UNB/MFA/TAOX; his4, Zeo* ^*R*^	*UNall*	*IL8*	a
pPNIc706	-*/PAOX/5′UTR/MFA/TAOX; his4, Kan*^*R*^ *Zeo*^*R*^		*IL8*	a
pPNic710	-*/PAOX/5′UTR/MFA/TAOX; his4, Kan*^*R,*^ *Zeo*^*R*^		*faeA*	a
pPNic957	*UNA1/PAOX/5′UTR/MFA/TAOX; his4, Kan* ^*R*^ *, Zeo* ^*R*^	*UNA1*	*faeA*	a
pPNic958	*UNA2/PAOX/5′UTR/MFA/TAOX; his4, Kan* ^*R*^ *, Zeo* ^*R*^	*UNA2*	*faeA*	a
pPNic959	-*/PAOX/UNB/MFA/TAOX; his4, Kan*^*R,*^ *Zeo*^*R*^	*UNB*	*faeA*	a
pPNic964	*UNA1/UNA2/PAOX/5UTR/MFA/TAOX; his4, Kan* ^*R*^ *, Zeo* ^*R*^	*UNA1 *+ *UNA2*	*faeA*	a
pPNic961	*UNA1/UNA2/PAOX/UNB/MFA/TAOX; his4, Kan*^*R*^, *Zeo*^*R*^	*UNall*	*faeA*	a
pPNy051	*UNA1/UNA2/PAOX/UNB/MFA/TAOX; his4, Kan* ^*R*^ *, Zeo* ^*R*^	–	–	a
pPNy063	*UNA1/UNA2/PAOX/UNB/MFA/TAOX; his4, Kan* ^*R*^ *, Zeo* ^*R*^	*UNall*[EAEA]	*faeA*	a
pPNy060	*UNA1/UNA2/PAOX/UNB/MFA/TAOX; his4, Kan* ^*R*^ *, Zeo* ^*R*^	–	–	a
pPNy059	*UNA1/UNA2/PAOX/UNB/MFA/TAOX; his4, Kan* ^*R*^ *, Zeo* ^*R*^	*UNall*[EAYVEF]	*faeA*	a

References: a: This study; b: Thermo Fisher

Structural elements: P*AOX*1: Promoter AOX1; 5′ UTR: 5′ Untranslated Region; UNA1, UNB and UNA2: regulatory element (see text); MFA: *S. cerevisiae* α-mating factor pre-pro leader sequence; T*AOX*; terminator *AOX*1; *his*4: *P. pastoris* histidine selection marker; *Kan*^R^: G418 resistance selection marker; *Zeo*^*R*^: *P. pastoris* zeocin selection marker

### Microliter scale screening of *P. pastoris* transformants

High throughput protein production screening of *P. pastoris* clones was performed in 2 mL deep-well plates (HJ-BIOANALYTIK-GmbH) filled with 800 µL BMGy or BMG. Each well was inoculated with one individual colony from a fresh selection plate. The cultures were grown under standard conditions (30 °C, 900 rpm, overnight) in an Infors HT microtron shaker. The optical density (OD) of the culture was measured at 600 nm. Protein production by methanol induction was started by transferring a volume amount of cells corresponding to a final 1.0 OD from an overnight preculture grown in BMG to a plate with methanol (BMM or BMMy). Every 24 h, a sample of 80 µL of culture was withdrawn, used for OD_600_ measurement and centrifuged at 2300×*g* for 2 min. Culture supernatant was collected and stored at − 20 °C until analysed for protein production yield. Methanol induced conditions were maintained by feeding the cells fed with methanol by addition of 80 µL of buffered minimal or complex medium containing 5% methanol (BMM5%, respectively BMMy5%). These fed batch protein production runs were carried out for 3 days (72 h).

### Enzyme activity assay of feruloyl esterase (FAE)

Throughout this studies the yield of *Aspergillus niger* feruloyl esterase A (FAE) recombinantly produced in *Pichia pastoris* GS115 was measured by its activity, thereby relating the amount of protein secreted into the production medium to the ability of the production medium to hydrolyse FAE-specific substrate, which requires proper posttranslational processing of the FAE (folding, glycosylation). Using enzyme catalysed hydrolysis of the substrate allowed to reproducibly compare the actual production yields requiring 10^3^–10^5^ times diluted production medium samples. FAE catalyses the hydrolysis of the ester bond in a variety of substrates. 4-Nitrophenyl ferulate (4-NPF) was used as the substrate throughout this study. Hydrolysis results into the formation 4-nitrophenol (4NP). The generation of this product was quantified by its absorbance at 405 nm. To prepare a 4-NPF stock solution 19 mg of 4-NPF (Taros Chemicals, Dortmund, Germany) was dissolved in 1 mL of DMSO. From this stock solution 20 μL were taken and added to 1980 μL of DMSO, generating a 0.6 mM 4-NPF stock. Finally, the 0.6 mM 4-NPF stock was diluted to 0.06 mM by addition of 18.0 mL Buffer (0.1 M potassium phosphate) or pre-diluted sample was used for the determination of the FAE activity. To the samples 190 μL of 37 °C pre-warmed 0.06 mM 4-NPF was added. The time dependent increase of the absorbance at 405 nm was measured using VICTOR^3^ multi label plate reader (PerkinElmer) at 37 °C with 5 min intervals for 1 h. As a reference, 0.3 and 0.9 mg/mL of FAE from *M. thermophile* (DuPont Industrial Biosciences) was taken along. Samples from *P. pastoris* GS115 that do not produce esterase were used as negative controls. One unit of FAE activity was defined as the amount of enzyme releasing 1 μmol of 4-nitrophenol from 4-NPF per min under the assay conditions. Reference experiments were carried out to relate the activity measurements with standard Bradford protein staining. They demonstrated that under the conditions 1 mg of FAE protein equals 19.6 U.

Controls were routinely taken along during analysis to exclude spontaneous hydrolysis of the substrate as well as non-enzymatic and non-specific hydrolytic activity in samples.

### Quantification of IL8

The amount of secreted IL8 was measured using a homogeneous bead based assay AlphaLISA (PerkinElmer), an ELISA-like assay using hIL8 specific antibodies. AlphaLISA assays were performed in white 384-well AlphaPlate (PerkinElmer #6004350) in a final volume of 20 µL. Samples were diluted either 10^−4^ or 10^−5^ in 1× AlphaLISA buffer (50 mM HEPES, pH 7.4, 0.1% casein, 1.0 mg/mL Dextran-500, 0.5% Triton X-100 and 0.05% Proclin-300). After incubation of the assay mix at room temperature for 3 h, the assay plate was read using an EnSpire multi label Alpha reader (PerkinElmer Inc.). A standard curve was plotted using the AlphaLISA counts versus the concentration of known IL8 standards. The concentration of IL8 in the samples were analysed according to a nonlinear regression using the 4-parameter logistic equation (sigmoidal dose–response curve with variable slope) and a 1/Y^2^ data weighting.

### Miscellaneous DNA techniques

All DNA manipulations were carried out according to standard methods [29]. DNA modifying enzymes were used as recommended by the supplier (NEB). DNA sequencing reactions were performed at BaseClear (Leiden, The Netherlands).

### Reference expression plasmids

Mature human *il8* (h-*il8*) DNA sequence was codon optimised for *P. pastoris* (GeneArt, Thermo Fisher Scientific, USA) and designed to be introduced as fusion protein with the *S. cerevisiae* α mating factor (MFA) secretion signal sequence. The synthetic DNA fragment was cloned using the *BamH*I and *Not*I sites of pPIC9Kα (Thermo Fisher Scientific, USA), resulting in plasmid pPNic384.

This expression vector was modified to enable its use for single copy integration using histidine selection as well as for the generation of multiple copy integrations using zeocin selection. The plasmid was digested with *Bgl*II and the fragment with the *His*4 selection marker, P*AOX1* and the kanamycin resistance marker was fused to the *Bam*HI and *Bgl*II fragment with the zeocin selection marker and a *E. coli* origin of replication from pPICZαA (Thermo Fisher Scientific, USA), resulting into plasmid pPNic706. The gene encoding *A. niger fae*A was codon optimised and synthesised by NZYtech, Lisbon, Portugal. It was cloned into plasmid pPNic706 using *Eco*RI and *Not*I, thereby replacing the IL8 sequence, resulting in plasmid pPNic710.

### Expression vectors with protein production enhancers and modified signal sequences

Expression vectors with different PPEs were derived from pPNic710. The different elements were introduced in this plasmid. The sequence of UNA1 was published by Xuan et al. [6]. This sequence consists of three tandem copies of the fragment from the position − 638 to − 510 bp in AOX1 promoter. To clone the UNA1 a synthetic fragment was made (Genewiz) which was extended at the 5′ site with the sequence for *Bgl*II and at the 3′ with the sequence 5′TTAA-PNF01-3′, in which PNF01=5′TTAAAACATCCAAAGACGAAAGGTTGAATGAAACCTTTTTGCCATCCGACATCCACAGGTCCATTCTCACACATAAGTG and which harbours an *Ale*I site.

UNA2 was designed by equipping the nucleotides 3411–4886 of *Cricetulus* sp. D63782.1 with a *Bgl*II site (at the 5′ end) and oligonucleotide PNF01. The DNA fragments with the UNA1 or UNA2 sequences bearing 5′*Bgl*II and 3′*Ale*I restriction sites were cloned in the expression vector pPNic710 (also digested with *Bgl*II and *Ale*I) resulting in expression vector pPNic957 and pPNic958, respectively. Combination of the UNA1 and UNA2 was designed by synthesis of 2077 bps DNA fragment with 5′*Bgl*II and 3′*Ale*I sites. The synthetic DNA fragment was cloned into pPNic710 digested with *Bgl*II and *Ale*I, thus resulting in expression vector pPNic964.

UNB (1149193–1149251, *Komagataella phaffii* GS115 chromosome 2, CP014716) was introduced using a synthetic construct in which the sequence was extended at the 5′ end with the AOX promoter sequence to insert the fragment in the *Blp*I site and at the 3′ end with α-mating factor (MFA) sequence harbouring a *Sfi*I site. A silent mutation was created in the AASSA peptide sequence of MFA to easily insert the *Blp*I–*Sfi*I fragment in the expression plasmid. The UNB fragment with 5′ *Blp*I and 3′ *Sfi*I restriction sites was cloned into expression vectors pPNic710 and pPNic964 resulting in expression vectors pPNic959 and pPNic961.

Plasmids with varying C-terminal spacer peptide sequences of MFA were constructed as follows. In order to construct the MFA sequence with spacer peptide EAEA, a 352 bps synthetic DNA fragment representing a part of MFA sequence containing EAEA spacer peptide with 5′ *Sfi*I, *Aar*I excision box and 3′ *Age*I was cloned in the expression vector pPNic961 digested with *Sfi*I, *Age*I, thus resulting in the plasmid pPNy051. A synthetic DNA fragment representing *Fae*A with *Aar*I restriction enzyme recognition sites on 5′ and 3′ ends were cloned in the plasmid pPNy051 thus resulting in expression vector pPNy063. MFA sequence with spacer peptide EAYVEF, a 335 bps synthetic DNA fragment representing a part of MFA sequence with EAYVEF spacer peptide with 5′ *Sfi*I, *Aar*I excision box and 3′ *Age*I was cloned in the expression vector pPNi961 digested with *Sfi*I, *Age*I, thus resulting in plasmid pPNy060. Expression vector pPNy059 was made by excising the *Fae*A from pPNic961 by restriction digestion with *Sna*BI and *Age*I and cloning in plasmid pPNy060 digested with *Sna*BI and *Age*I.

### *IL*8 expression vector with protein production enhancers (PPEs)

The zeocin selection marker from pPICZαA (Thermo Fisher Scientific, USA) was isolated by initially digesting the vector pPICZαA with *Bam*HI. The 5′ and 3′ ends were made blunt ended using DNA polymerase I. Further, on the *Bam*HI linearized DNA was digested with *Pci*I resulting in 1187 bps zeocin selection marker. This fragment was used as insert to be ligated into expression vectors pPNic384 and pPNic775 digested with *Sbf*I, blunted with DNA pol I, restriction digested using *Pci*I resulted in expression vector pPNic948, pPNic947.

### Bioreactor fermentations

Bioreactor fermentations were performed in a Biostat UD-30 stainless steel reactor (B. Braun Biotech International, Melsungen, Germany) with a total volume of 42 L (28 cm inner diameter and 71 cm height), a maximum working volume of 30 L and a d/D value relation (relation of stirrer diameter to vessel diameter) of 0.375. The stirrer was equipped with three stirring blades consisting of six paddles each. Probes for measuring dissolved oxygen (dO_2_) (model 25; Mettler Toledo GmbH, Steinbach, Switzerland), pH (model Pa/25; Mettler Toledo GmbH), foam (model L300/Rd. 28; B. Braun Biotech International), temperature (pt 100 electrode; M. K. Juchheim GmbH, Fulda, Germany), and optical density at 850 nm (OD_850_) (model CT6; Sentex/Monitek Technology Inc.) were inserted into the probe ports of the bioreactor. Primarily, foam was controlled automatically by a Funda-Foam mechanical foam destroyer (B. Braun Biotech International, Melsungen, Germany) or secondarily by the manual addition of low amounts of a 25% (v/v) Silfoam SE 2 (Wacker Chemie AG, Nünchritz, Germany) antifoam emulsion with water. The fermentation parameters were controlled and recorded by a digital control unit in combination with a MFCS/win software package (B. Braun Biotech International).

Primary and secondary pre-cultures for fermentations were prepared, optimal secondary pre-culture inoculation OD_600_ was calculated and main culture inoculation was done essentially as described by Tolner et al. [30]. For pre-culture growth, however, Buffered Minimal Glycerol (BMG) medium (100 mM potassium phosphate (pH 6.0), 1.34% (w/v) YNB, 0.02% (w/v) biotin) containing 2% (v/v) glycerol was used. Main fermentations were carried out in 16 L Basal Salts Medium (BSM) consisting of (per L) 26.7 mL 85% H_3_PO_4_, 0.93 g CaSO_4_, 18.2 g K_2_SO_4_, 14.9 g MgSO_4_ × 7H_2_O, 4.13 g KOH, and 40.0 g glycerol, which was supplemented with 4.35 mL/L of *Pichia* Trace Metals solution (PTM_1_; per L: 6.0 g CuSO_4_ × 5H_2_O, 0.08 g NaI, 3.0 g MnSO_4_ × H_2_O, 0.2 g Na_2_MoO_4_ × 2H_2_O, 0.02 g H_3_BO_3_, 0.5 g CoCl_2_, 20.0 g ZnCl_2_, 65.0 g FeSO_4_ × 7H_2_O, 0.2 g biotin and 5.0 mL H_2_SO_4_). 25% (v/v) NH_4_OH solution was used for adjusting the fermentation medium to pH 5.7 and for maintaining pH during the fermentation run.

Main cultures were inoculated with secondary pre-cultures of 5% (v/v) of the initial fermentation volume and were subsequently grown in fed-batch mode at 30 °C. After batch glycerol was consumed, visible by a spiking of the dO_2_ value after 22–26 h, the cultures were fed with in total 5% (v/v) of the initial fermentation volume 50% (v/v) glycerol supplemented with 12 mL/L PTM_1_ for another 8 h (approx. 7 g/h/L). During glycerol feed dO_2_ was kept at 20% by automatic regulation of the stirrer rpm (100–650 rpm) and manually adjusted aeration rates of 0.625–3.125 vvm. Two hours before the fed glycerol was depleted, 0.2% (v/v) of the initial fermentation volume methanol containing 12 mL/L PTM_1_ was added to the remaining feed glycerol to slowly induce the AOX1 promotor enabling methanol degradation and recombinant protein expression. After the fed-glycerol/methanol mixture was completely consumed as well (dO_2_ spiking) the cultures were switched to methanol-only feed (containing 12 mL/L PTM_1_). The initial feeding rate of approximately 3.5 mL/h/L was maintained for about 8–10 h and subsequently increased to about 6.5 mL/h/L. The actual feeding rate also modified to maintain a dO_2_ value of 10–20% and to limit foam formation. Fermentations were continued during 144–150 h (i.e. for 114–120 h of methanol induction).

During fermentation samples were withdrawn at regular intervals to collect 5 mL of cell free supernatant for protein quantification by ELISA and for determination of OD_600_ and cell fresh weight (CFW). For the latter samples were acidified with 10 M HCl to pH 3 to dissolve precipitated salts. Cell pellets obtained by centrifugation at 5000×*g* for 5 min were frozen at − 20 °C and subsequently freeze dried for 24 h to determine the cell dry weight (CDW).

Growth rates and methanol feed rates were calculated for the time periods between two points of measurement by dividing the increase of CDW or the amount of methanol fed, respectively, by the elapsed time since the last measurement.
